# Decision Making under Risk Condition in Patients with Parkinson’s Disease: A Behavioural and fMRI Study

**DOI:** 10.3233/BEN-2010-0277

**Published:** 2010-11-23

**Authors:** Kirsten Labudda, Matthias Brand, Markus Mertens, Isabelle Ollech, Hans J. Markowitsch, Friedrich G. Woermann

**Affiliations:** ^1^Department of Physiological PsychologyUniversity of BielefeldGermany; ^2^MRI UnitMara HospitalBethel Epilepsy CenterBielefeldGermany; ^3^General Psychology: CognitionUniversity of Duisburg-EssenGermany; ^4^Erwin L. Hahn Institute for Magnetic Resonance Imaging, EssenGermany; ^5^Institute for Advanced StudyAlfried-Krupp WissenschaftskollegGreifswaldGermany

**Keywords:** Executive functions, dopamine, prefrontal cortex, anterior cingulate cortex, parietal lobe, risk

## Abstract

We aimed to study whether previously described impairment in decision making under risky conditions in patients with Parkinson's disease (PD) is affected by deficits in using information about potential incentives or by processing feedback (in terms of fictitious gains and losses following each decision). Additionally, we studied whether the neural correlates of using explicit information in decision making under risk differ between PD patients and healthy subjects. We investigated ten cognitively intact PD patients and twelve healthy subjects with the Game of Dice Task (GDT) to assess risky decision making, and with an fMRI paradigm to analyse the neural correlates of information integration in the deliberative decision phase. Behaviourally, PD patients showed selective impairment in the GDT but not on the fMRI task that did not include a feedback component. Healthy subjects exhibited lateral prefrontal, anterior cingulate and parietal activations when integrating decision-relevant information. Despite similar behavioural patterns on the fMRI task, patients exhibited reduced parietal activation. Behavioural results suggest that PD patients’ deficits in risky decision making are dominated by impaired feedback utilization not compensable by intact cognitive functions. Our fMRI results suggest similarities but also differences in neural correlates when using explicit information for the decision process, potentially indicating different strategy application even if the interfering feedback component is excluded.

## 1. Introduction

Some studies report higher prevalence of impulsive and risk-seeking behaviour in patients with PD, e.g. reflected in higher rates of pathological gambling [1], risky driving behaviour [2] or hypersexuality [3] compared to the normal population. The phenomenon of risk- and reward-seeking behaviour has been discussed as a result of dopaminergic treatment. These behavioural phenomena might be more strongly related to dopamine agonist treatment than to levodopa therapy [1,4–6]. However, Avenzi et al. [2] described two PD patients who developed pathological gambling and stopped gambling after a reduction of levodopa dosage. In accordance with these behavioural observations, recent neuropsychological studies have also objectified deficits in experimental decision making tasks that demand the processing of reward and punishment [7–10]. The most frequently used task to assess this kind of decision making – termed decision making under ambiguity or feedback-based decision making – is the Iowa Gambling Task (IGT) [11,12]. This computerized card gambling task has implicit rules for gains and losses associated with four different card decks. Initially, subjects have to figure out advantageous and disad-

ISSN 0953-4180/10/$27.50 © 2010 – IOS Press and the authors. All rights reserved vantageous options by using feedback (gains and losses) received after each card selection. The assumption that disadvantageous IGT performance in PD patients is the result of reductions in processing and utilizing emotional feedback is in line with the recent finding of reduced skin conduction responses (frequently interpreted as a marker of affective responsiveness) in this patient group during IGT performance [7,13].

In a previous study, we also observed deteriorations in decision making under risk conditions in patients with PD [14]. In this study, the Game of Dice Task (GDT, [15]) was used. GDT performance in PD patients with and without additional pathological gambling was also assessed in a recent study by Rossi et al. [16]. The authors reported that PD patients with and without gambling problems did not differ in their GDT performance but, unfortunately, the author did not provide GDT netscores. Thus, it remained unclear whether or not the patients exhibited risky decision making. In contrast to the IGT, this gambling task provides explicit information about the gains and losses and the winning probabilities. By explicitly providing information about consequences and their probabilities, subjects are enabled to plan their task performance. Previous studies revealed reductions of performance in this task in several groups of patients with neurological or psychiatric disorders, such as patients with bilateral amygdala damage, Alzheimer's disease, Korsakoff's disease, pathological gambling, bulimia nervosa and others [15, 17–21]. These studies also indicate that GDT performance is based on a cognitive/deliberative component, i.e. mainly executive functions that are relevant for categorizing options according to gains and losses and their probabilities, developing and maintaining a long term decision strategy and monitoring, as well as modifying performance in the task's course. Furthermore, task performance is influenced by an affective component, i.e. processing feedback in terms of gains and losses to modify decision making (see [22,23]). Our previous study on risky decision making in PD patients has shown that the patients' GDT performance is associated with both components: GDT performance correlated with executive functioning and the utilization of feedback following a decision. This interpretation is also in line with a recent study by Euteneuer et al. [13] who report correlations between decision making deficits on the GDT and executive functions. Furthermore, PD patients in this study also showed reduced SCRs after receiving negative feedback, indicating the impact of emotional feedback processing on the GDT performance. However, to date it remains unclear which of the two components mainly dominates risky decision making in PD patients. We therefore aimed to study decision making under risk conditions in PD patients without executive and other cognitive disturbances in the current study, to assure that potential deficits cannot be due to deteriorations of the cognitive component. Beyond the original GDT, we used an additional fMRI task that is based on the GDT but does not include the feedback component [24,25]. On a behavioural level, if cognitive (e.g. executive) functions dominate risky decision making, one would expect that our sample of high-functioning PD patients is unimpaired on both, the fMRI task without feedback and the GDT with feedback. If feedback processing dominates decision making, one would expect selective impairments on the GDT but not on the fMRI task. According to previous results on altered reward processing and decision making in PD patients (see above), we assume that the given feedback might be sufficient to impair decision making despite intact executive functions and, thus, we hypothesize selective disturbances on the GDT. Frank and coworkers demonstrated that PD patients on medication are able to learn from positive but not from negative feedback in cognitive procedural learning tasks [26,27]. We therefore aimed to analyse whether the patients are equally disturbed in processing negative and positive feedback or whether feedback processing is selectively altered in the context of decision making. The use of the fMRI task further enables us to study possible alterations of the neural correlates associated with the cognitive component of decision making under risk. We recently demonstrated that this deliberative component of the risky decision making process is associated with structures discussed as relevant for executive functions such as the dorsolateral prefrontal cortex and the parietal lobe [25]. We also found activations within the anterior cingulate gyrus that were assumed to be due to conflict detection between concurring decision options.

Neuroimaging studies using cognitive tasks in PD patients have delivered inconclusive results with regard to task relevant – e.g. prefrontal – activation in PD patients. With respect to the lateral prefrontal cortex, some findings suggest increased activity in high-level cognitive tasks – at least if patients have deficits on the behavioural level [28]. Other neuroimaging studies reported frontolateral hyper- as well as hypoactivation [29–31]. Further studies suggest activation decreases within the dorsolateral prefrontal cortex in PD patients [32,33] but only in patients in the 'off' state (e.g. [34]). Results of Fera et al. indicate that medicated, non-demented PD patients show patterns of lateral prefrontal activation very similar to those of healthy subjects when performing tasks assessing executive functions [34]. With respect to frontomesial activations, results from neuroimaging studies using executive tasks, such as the Wisconsin Card Sorting Test [29, 35] but also reward-based tasks [36] revealed increased activation within this prefrontal brain region compared to healthy subjects.

There is only one study on the neural correlates of decision making in PD patients. Using FDG PET, Thiel et al. demonstrated reduced frontomesial activation and thalamic deactivation in patients when performing the IGT [37]. Only the healthy subjects showed activation within the mesial prefrontal, orbitofrontal and anterior cingulate cortex. Dorsolateral prefrontal activity was comparable in PD patients and healthy subjects.

In the current study, we only investigated cognitively intact patients on dopaminergic medication (dopamine agonist and/or levodopa) and of the akinetic-rigid type. We therefore hypothesized that PD patients are unimpaired on the behavioural measures of the fMRI task and assume that their lateral prefrontal activation pattern might also be similar to those of the control subjects. However, according to the neuroimaging studies mentioned above, we assume that the patients might show reduced activity within the anterior cingulate gyrus, a region that has been shown to be crucially involved in the fMRI task used in this study in healthy subjects [25]. Beyond its general role in processing uncertainty [38], this region seems to be stronger associated with the selection of an option compared to the anticipation of feedback [39], is discussed as relevant for detecting conflicts between concurring decision options [40] and has been demonstrated to be activated when choosing risky alternatives [41].

## 2. Methods and subjects

### 2.1. Participants

We investigated 12 patients with PD recruited from a local support group and/or from a practice-based neurologist specialized on PD. One patient was excluded from the original sample due to technical problems that led to missing behavioural data during the fMRI task. Another patient was excluded due to a major depression diagnosis. Thus, ten right-handed PD patients (8 males) finally constituted the PD group. Exclusion criteria were current psychiatric comorbidity, neurological disorders other than PD, medical treatment with anticholinergic medication and/or other psychotropic substances. To assure that participants had no signs of cognitive impairment or dementia, we excluded participants with a DemTect transformed score $< 13$ (according to the test cut-off proposed by Kessler et al. [44]) and t-scores of $< 40$ on the Modified Card Sorting Test measuring executive functions (see below). All patients were treated with typical dopaminergic medication (levodopa, dopamine agonists), seven patients were additionally medicated with MAO-B inhibitors and/or NMDA agonists. According to the criteria of Hoehn and Yahr [42], most of the patients were classified as stage III (median = 3, range 2–4) and mean duration of illness was 84.75 months (SD = 44.49, range: 24–144 months). To avoid movement artifacts within the fMRI data, we only recruited patients of the akinetic-rigid type.

Additionally, a comparison group (CG) with 12 healthy control subjects (6 males, all right-handed) was investigated with the neuropsychological test battery and the decision making fMRI task. Imaging data of the healthy subjects, but not the neuropsychological results, were reported previously [25]. Patients and CG subjects did not differ according to gender distribution ($Chi^2 = 2.12$, df = 1, $p = 0.20$[1]), age (PD patients: mean = 57.60, SD = 7.83; CG subjects: mean = 62.33, SD = 4.81, $t = 1.74$, df = 20, $p = 0.10$) and years of education (PD patients: mean = 10.20, SD = 2.10; CG subjects: mean = 10.92, SD = 1.78, $t = 0.87$, df = 20, $p = 0.40$). All participants gave written informed consent prior to the investigation. Neither control subjects nor patients received financial compensation for participation. The study was approved by the local ethic commission.

### 2.2. Neuropsychological assessment

The neuropsychological test battery comprised standardized tests. To exclude subjects with signs of general cognitive impairment, the DemTect [44], consisting of subtests for direct and delayed verbal learning, number transcoding, verbal fluency and working memory, was used. We administered the subtest 'reasoning' from a German intelligence test battery (Leistungsprüfsystem, [45]) to estimate subjects' IQs. Verbal learning and memory were measured with the California Verbal Learning Test (CVLT [46]), a word list

[1] Although not significant, gender distribution slightly differs in the two groups. However, a number of studies did not find any impact of gender on GDT performance [43].

learning task with an additional delayed recall trial. Short-term and working memory were assessed with the revised Wechsler Memory Scale subtests digit and block span forward and backward [47]. In addition, we employed three tests to measure executive functions: The Modified Card Sorting Test (MCST, [48]) for the assessment of organisation, cognitive flexibility and set-shifting, the Word Colour Interference Test [49] and the Trail Making Test for the assessment of inhibition, interference susceptibility, and cognitive flexibility. Results of the neuropsychological test battery are summarized in Table 1. For a detailed description of the neuropsychological tasks see Lezak [50] and Spreen and Strauss [51].

### 2.3. *Game of dice task*

We used the computerized Game of Dice Task (GDT, [15]) to assess decision making under risk conditions. In this task, a single die was thrown 18 times. Subjects were instructed to maximize a fictitious starting capital (1,000 €[2]) by guessing which number would be thrown before the die is rolled. Subjects can choose between different options associated with defined gains/losses: they can select one specific number of the die (winning probability 1:6, associated gain/loss 1,000 €), a combination of two numbers ('1, 2' or '3, 4' or '5, 6'; winning probability 2:6, associated gain/loss 500 €), a combination of three numbers ('1, 2, 3' or '4, 5, 6'; winning probability 3:6, associated gain/loss 200 €), or a combination of four numbers ('1, 2, 3,4' or '2, 3, 4, 5' or '3, 4, 5, 6'; winning probability 4:6, associated gain/loss 100 €). Subjects received an associated amount of money (1,000, 500, 200 or 100 €) when one of the numbers of a combination chosen was thrown with the single die. In the event that a number not contained in the selected combination was thrown, subjects lost the same amount of fictitious money. The amounts of gains and losses were linked to the winning probabilities, i.e. high potential gains/losses were associated with low winning probabilities and low gains/losses were associated with high winning probabilities (e.g. the choice of one number was linked with 1,000 € gain/loss; the choice of a combination of four dice was associated with 100 € gain/loss). After each roll of the die, a visual and acoustic signal indicated whether the subject had won or lost, and the gained or lost sum was added to or subtracted from the current balance.

In order to analyse task performance, we categorized two out of the four alternative categories as risky, or disadvantageous, (one single number and combinations of two numbers), because winning probabilities were lower than 34%. Thus, a frequent selection of these options would lead to a negative outcome in the long run. The selection of the combinations of three or four numbers was considered non-risky, or advantageous, because winning probabilities were higher than 50%. Furthermore, we analyzed the use of negative feedback in the GDT. Feedback was evaluated as used when a subject lost 500 or 1,000 € after the selection of one of the risky options and subsequently selected a non-risky option before the next die was rolled. As the number of negative feedback differs between subjects, the percentage of negative feedback use was calculated for all subjects that received negative feedback at least one time.

### 2.4. *fMRI paradigm*

## 3. Stimulus presentation and design

The stimuli presented in the blocked fMRI paradigm are based on the GDT, but the feedback phase was completely removed to avoid potential confounds of the neural correlates underlying the cognitive-deliberative phase and the feedback phase [24,25]. The fMRI task had one activation condition ($A$), one high-level control condition ($B$) and a low-level control condition ($C$). The conditions are described in detail below. A baseline condition that required the fixation of a moving cross was presented between conditions $A$, $B$ and $C$ to avoid carryover effects of activation between conditions. For stimulus presentation and response acquisition, *Presentation* software (Neurobehavioral Systems, Albany, CA, USA) was used on a laptop computer. The stimuli were displayed onto a translucent screen, watched through a mirror attached to the inside of the head coil.

Conditions $A$, $B$ and $C$ were separated into six blocks, each containing six items (36 items per condition), that were shown in random order to the subjects. In each block, stimuli were presented for five seconds (6 items x 5 seconds = 30 seconds per block). The baseline condition was presented for 30 seconds between each block of conditions $A$, $B$ and $C$. Altogether, 18 activation blocks and 19 baseline blocks were performed by each subject. Prior to the fMRI proce-

[2]Using fictitious instead of real gains and losses is common in neuroscientific decision making research. This method was used in numerous studies using the GDT and other gambling tasks (see e.g. review by Dunn, Dalgleish and Lawrence [52]).

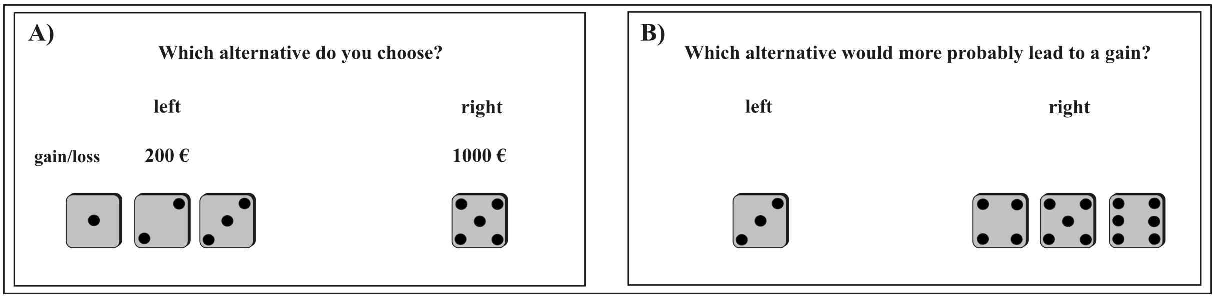

Fig. 1. Examples of stimuli used in condition A (1A) and in the high-level control condition B (1B).

dure, subjects passed a test session to assure that the introductions were understood and that subjects were able to make their decisions within the given time interval. Subjects were particularly briefed that they would not receive any feedback, i.e. that the depicted incentives in condition A would not be paid-off during or after the scanning session. The activation and control conditions are described in the following section.

### *3.1. Condition A*

In this condition, two combinations of dice were presented on the screen. Each dice arrangement was linked to a specific gain/loss (see Fig. 1A). According to the GDT, a single die was associated with 1,000 € gain/loss, a combination of two dice with a gain/loss of 500 €, an arrangement containing three dice with 200 € gain/loss and combinations of four dice were always presented with a potential gain/loss of 100 €. Subjects were instructed to imagine that a single die was thrown. They were told that the amount of money presented above each dice arrangement would be theoretically received, if the number fictitiously thrown was included in the chosen combination. Subjects were also told that they would lose the same amount of money if the thrown number was not contained in the chosen arrangement. Participants were explicitly briefed that no die would actually be thrown and that they, therefore, would not receive feedback after a decision. They were instructed to always choose one of the two arrangements presented and to behave like being in a real gambling situation. Subjects should follow their personal preferences. Decisions were made by a button press (right thumb for choosing arrangements on the right side of the screen and left thumb for choosing arrangements on the left side) and were recorded with *Presentation* software.

### *3.2. Condition B*

Analogous to condition A, two arrangement of dice,each containing different numbers of dice (1 to 4), were presented on the screen (see Fig. 1B). In contrast to condition A, no potential gains/losses were indicated. Subjects were again told to imagine that one single die would be thrown. In this condition, participants always had to indicate, by a button press, which of the two dice arrangements had a higher winning probability. Winning probability was defined as the probability of reaching congruency between a virtually thrown number and one of the numbers of the chosen dice arrangement. The arrangements presented were analogous to those of the GDT (see above). In the instruction, it was again emphasized that it was not indicated whether the subjects' decisions were right or wrong after a decision was made.

### *3.3. Condition C (low-level control condition)*

Again, two arrangements of dice were shown on the screen and, analogous to condition $B$, no incentives were announced. One of the two arrangements always contained a blank die. Subjects were instructed to indicate the side of the blank die via button pressing. This condition was added because visual input and required motor activity was comparable to the other conditions of the task used, but no information about probabilities and incentives had to be processed.

## 4. Data acquisition

Functional MRI scanning was performed with a 1.5 Tesla scanner (Siemens Magnetom Symphony, Erlangen, Germany) equipped with echo planar imaging (EPI) capability and a standard head coil. Scout T1-weighted images were obtained in every subject before the fMRI procedure to position the axial T2*-weighted images along the anterior commissure-posterior commissure (AC-PC) line. To exclude subjects with gross brain pathology and to provide anatomical references,

Table 1
Results of the neuropsychological assessment for the PD patients and the CG

| Domain/test | Value | PD patients Mean (S.D.) | CG Mean (S.D.) | p |
|---|---|---|---|---|
| **General cognitive abilities** | | | | |
| Subtest 'reasoning' (LPS 4) | | | | |
| Estimated IQ | | 110.80 (14.19) | 108.73 (10.96) | 0.71 |
| DemTect | | | | |
| Transformed score | max = 18 | 16.10 (2.13) | 16.83 (1.64) | 0.37 |
| **Memory** | | | | |
| California Verbal Learning Test | | | | |
| Learning (sum of trial 1–5) | per | 61.80 (25.51) | 54.92 (29.14) | 0.57 |
| Recall short delay (trial 7) | per | 39.40 (28.69) | 37.75 (29.91) | 0.90 |
| Recall long delay (trial 8) | per | 50.40 (28.96) | 55.33 (33.76) | 0.72 |
| Wechsler Memory Scale – Revised | | | | |
| Digit span forward | per | 59.50 (30.37) | 61.17 (24.05) | 0.89 |
| Digit span backward | per | 55.30 (26.66) | 56.83 (29.53) | 0.90 |
| Block span forward | per | 50.60 (27.45) | 34.33 (28.00) | 0.19 |
| Block span backward | per | 65.20 (31.00) | 56.50 (29.68) | 0.52 |
| **Information processing and executive functions** | | | | |
| Word Colour Interference Test | | | | |
| Reading words | per | 69.90 (22.40) | 85.42 (11.65) | 0.05 |
| Naming colours | per | 70.70 (17.75) | 81.25 (14.06) | 0.14 |
| Interference trial | per | 73.20 (19.95) | 82.33 (8.48) | 0.20 |
| Modified Card Sorting Test | | | | |
| Categories | t-score | 49.90 (7.13) | 52.17 (5.81) | 0.98 |
| Errors | t-score | 47.30 (3.56) | 47.25 (4.90) | 0.91 |
| Perseverations | t-score | 53.80 (5.03) | 53.58 (3.40) | 0.42 |
| Semantic verbal fluency | RS | 26.60 (5.42) | 29.50 (5.58) | 0.23 |

S.D. = Standard deviation.
max = maximum.
per = percentiles.
sec = seconds.
LPS = Leistungsprüfsystem (German intelligence test battery; Sturm, Willmes & Horn, 1993).
RS = raw scores.

a T1-weighted 3D-sequence was obtained from all subjects prior to the fMRI procedure using magnetisation-prepared, rapid acquisition gradient-echo (MPRAGE, TR = 11.1 ms, TE = 4.3 ms, slice thickness 1.5 mm, FOV 201 × 230 mm, matrix 224 × 256).

For fMRI, 10 sets of 20 axial T2*-weighted MR slices of 5 mm thickness were obtained using a standard EPI sequence during each block. The following scan parameters were used: TR = 3,000 ms, TE = 40 ms, FOV = 192 mm, flip angle = 90°, matrix = 64 × 64. Axial slices were oriented along the anterior AC-PC line and covered the whole brain.

## 5. Image analysis

Functional MRI data were analyzed by using MATLAB and Statistical Parametric Mapping (SPM5, Wellcome Department of Imaging Neuroscience, London, UK; http://www.fil.ion.ucl.ac.uk/spm) for all imaging pre-processing and voxel-based statistical analyses within the context of general linear model. The T2*-weighted images were realigned using the default SPM5 algorithm to correct for subjects' movements. Spatial normalization, to reduce anatomical differences before group comparison, was conducted by again using default settings and the standard stereotactic space of SPM5, i.e. the Montreal Neurological Institute (MNI) brain. Spatial smoothing followed with a Gaussian kernel (10 mm full width at half maximum) to increase signal and anatomical conformity. A fixed-effect analysis on a voxel-by-voxel basis was carried out for individual subjects (threshold $p < 0.001$, uncorrected) and contrast images were created for the different task conditions. A second level t-test (random-effects) analysis was conducted to identify significant differences of the BOLD response within the planned linear contrast for the whole group. T-statistics were corrected for multiple comparisons at p=.05 and the minimum size of displayed clusters was 10 voxels. The MNI coordinates of the major activations were transformed into the Talairach and Tournoux space [53] using a correction procedure [54] and subsequently fed into the Talairach Daemon [55] to obtain anatomical projections of maximum activation.

## 6. Statistical analysis

All variables were tested for normal distribution with the Kolmogorov-Smirnov-Test separately for the patient and comparison group. No significant deviations from the normal distribution were revealed for all neuropsychological variables (all $p \geqslant 0.07$), as well as for the IGT and GDT scores (all $p \geqslant 0.19$). Thus, we used parametric methods (t-tests for independent samples) for all analyses of neuropsychological and sociodemographic data.

## 7. Results

### 7.1. Neuropsychological assessment

The results of the neuropsychological test battery are summarized in Table 1. Results indicate that the PD patients' performance was entirely within the normal range in all domains studied and that patients did not differ significantly from the CG subjects.

### 7.2. GDT performance

Patients' performance differed significantly from that of the healthy subjects: PD patients selected the risky options (one die or two dice) more frequently than the healthy subjects did (PD patients: mean = 10.30, SD = 5.27; CG subjects: mean = 5.67, SD = 4.79, $t = -2.16$, df = 20, $p = 0.04$). PD patients used negative feedback less frequently compared to the CG subjects (i.e. the selection of a non-risky option following a loss due to the selection of a risky option).[3] PD patients only changed to a non-risky option after a loss due to a risky selection in 36.45% (SD = 36.24) whereas CG subjects used 82.32% (SD = 33.22) of the negative feedback received after a risky decision and an accordant loss ($t = 3.03$, df = 19, $p = 0.007$). PD patients and healthy subjects did not differ according to the percentage of used positive feedback following a non-risky selection (100 or 200 € gain) in order to again select a non-risky option in the next trial (PD patients: mean = 63.14%, SD = 33.05; CG: mean = 75.51%, SD = 20.85; $t = 1.00$, df = 17, $p = 0.33$).

[3] As one of the healthy subjects did never receive negative feedback after the selection of a risky option, feedback analysis is based on the data of 11 CG subjects and 10 PD patients.

### 7.3. Behavioural results of the fMRI task

Behavioural data of the fMRI paradigm are similar in both groups. In condition A, both groups did not differ according to the number of selections of the non-risky options (PD patients: mean = 91.57%, SD = 11.06, CG subjects: mean = 91.29%, SD = 8.26, $t = 0.08$, df = 20, $p = 0.94$). In condition B, both groups did not differ according to the frequency of selections of those dice arrangements with a higher winning probability (PD patients: mean = 97.77%, SD = 3.42, CG subjects: mean = 97.64%, SD = 4.25, $t = 0.07$, df = 20, $p = 0.95$). In the low level control condition C, both groups reliably identified the blank die (PD patients: mean = 97.72%, SD = 5.35, CG subjects: mean = 98.79%, SD = 2.01, $t = 0.64$, df = 20, $p = 0.53$).

### 7.4. Imaging results

As a first step, we analysed activation patterns in the contrasts of interest within both groups separately. We subtracted BOLD responses in condition B (information about probabilities only) from those of condition A (information about probabilities and incentives) to objectify activations associated with the integration of information about probabilities and incentives compared to those associated with the processing of probabilities only (A > B). Furthermore, we subtracted activations associated with the low level control condition from those of condition A (A > C). Within the PD group, we did not find significant activation patterns within all contrasts analysed (A > B, A > C) and within the inverted contrasts (A < B, A < C; one sample t-tests, all p corrected > 0.49).

Results of the healthy subjects are described in detail elsewhere [25]. In short, main results of the contrast A > B (second level analysis, one sample t-test; all corrected $p < 0.05$) revealed significant activations within the dorsolateral prefrontal cortex (DLPFC) bilaterally (= 50, $y = 32$, $z = 24$, $k = 1459$, $Z = 4.62$; $x = -34$, $y = 10$, $z = 46$, $k = 210$, $Z = 4.35$), within the right anterior cingulated gyrus ($x = 2$, $y = 28$, $z = 36$, $k = 1526$, $Z = 4.90$), within the left parietal lobe ($x = -48$, $y = -58$, $z = 42$, $k = 182$, $Z = 4.35$) and the right precuneus ($x = 4$, $y = -74$, $z = 54$, $k = 180$, $Z = 3.80$), as well as within the occipital lingual gyrus ($x = 6$, $y = -90$, $z = -14$, $k = 169$, $Z = 4.24$). Within the contrast of the conditions A > C, we found significant activations within the left and right DLPFC ($x = 46$, $y = 34$, $z = 24$, $k = 918$, $Z = 4.94$; $x = -52$, $y = 18$, $z = 26$, $k = 190$, $Z = 3.67$), the supramarginal gyrus ($x = 42$, $y = -46$, $z = 38$, $k = 733$, $Z = 3.96$) and the right cerebellum (declive of vermis; $x = 2$, $y = -72$, $z = -29$, $k = 436$, $Z = 4.08$).

We then compared activation patterns of the CG and the PD group. The only significant group difference was found within the contrast A $>$ C. Healthy subjects showed significantly stronger activations within the right inferior parietal lobe than PD patients did ($x = 42$, $y = -44$, $z = 40$; $k = 222$, $Z = 3.83$, p corrected $= 0.001$, see Fig. 2).

PD patients did not exhibit stronger activations in any contrast compared to the CG (all p corrects $> 0.80$). In order to test whether there was a condition x group interaction, we calculated a two-way ANOVA with condition (A $>$ C, B $>$ C) and group as independent variables. We did not find a significant interaction even at a low uncorrected threshold of $p < 0.05$.

We additionally analysed potential differences within the PD group and between both groups in the contrasts of interest (A $>$ B, B $>$ C) in anatomically defined regions of interest (ROI, using the WFU Pickatlas Tool 1.03; http://fmri.wfubmc.edu/cms/software#WFU_Pick Atlas, ANSIR Laboratory, Department of Radiology, Wake Forest University School of Medicine, Winston-Samel, North Carolina, USA). We did not find further within or between group BOLD differences in a lateral prefrontal ROI, in an anterior cingulate gyrus ROI, in a ventral striatum ROI and in a ROI including putamen, pallidum and caudate nucleus.

## 8. Discussion

Behavioural results of the present study confirm our previous findings of impaired decision making under risk in non-demented patients with PD using the GDT. In a decision making situation that provides explicit information about consequences and probabilities and fictitious monetary reward and punishment, PD patients chose the risky options, i.e. options with high luring gains but low winning probabilities, more frequently compared to healthy subjects. The additional feedback analysis reflected that PD patients' ability to use negative feedback after a risky decision is strongly reduced. Nevertheless, as hypothesized, on the decision making fMRI task that did not include the feedback aspect of the GDT, we did not find any behavioural difference between the PD patients and healthy subjects. Therefore, we assume that PD patients are not generally impaired in the cognitive processing of the information relevant for their decisions – at least in those patients with intact cognitive and executive functions. Our results rather suggest that reductions in reward and punishment processing are sufficient to deteriorate the whole decision process in PD patients even when cognitive functions are intact. The behavioural fMRI results further exemplify that – in contrast to receiving reward and punishment – the presentation of potential incentives alone does not lead to disadvantageous decision making. In contrast to PD patients, healthy subjects benefit from receiving feedback. Brand [23] has shown that in an experimental GDT version without feedback, healthy subjects had a higher frequency of selecting risky options compared to the original version. Thus, providing feedback in healthy subjects leads to a moderate decrease of risky decisions. In PD patients, providing feedback leads to an increase of risky decisions whereas decision making without feedback is normal. This disadvantageous decision making seems to be specifically due to an impairment of using negative feedback. In accordance with the results of Frank et al. [26,27], impairments of feedback processing were selective in the present study: PD patients did not use negative feedback (in terms of losses) to modify their behaviour. In contrast, they did not differ from the healthy subjects according to the use of positive feedback (in terms of gains) to stick to the non-risky options in the next trial. Our results thus support the view that decision making deficits in PD patients might be due to impairments of the so-called limbic loop, linking the mesial orbitofrontal cortex and the anterior cingulate gyrus to the ventral striatum [56,57]. This loop has been demonstrated to be involved in feedback processing in a number of studies and was assumed to be affected already in early PD [37]. However, in the recent study by Euteneuer et al., the authors attribute a reduced GDT performance to dysfunctions of the so-called dorsolateral prefrontal cognitive loop [13]. This loop mainly comprises the dorsolateral prefrontal cortex, the lateral orbitofronatal cortex and the striatum and is associated with executive dysfunctions in PD patients [58]. In contrast to our study, the patients in the study of Euteneuer and colleagues had reductions in executive functions. However, the authors also assume deteriorations of the limbic loop to some degree, as their patients also showed altered SCRs, linked to deficient negative feedback processing. Taken together, our previous results [14] and those of Euteneuer suggest that impairments of both, the cognitive and the limbic loop may cause risky decision making impairments. Our current results extend these findings by showing that

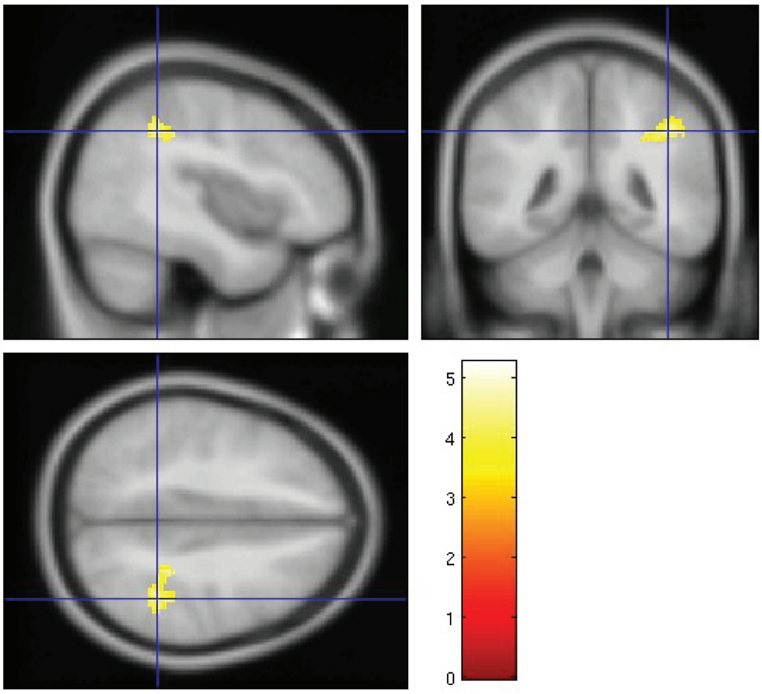

Fig. 2. Significant BOLD increases in the healthy subjects compared to the PD patients in the contrast A > C ($p = 0.001$, corrected).

deficits in negative feedback processing are sufficient to cause decision making problems, despite intact cognitive functioning in PD patients.

Our fMRI results further support the interpretation of an intact cognitive loop in our PD patients, as we did not find significant activation differences within the PFC between healthy subjects and PD patients while integrating information provided for the decision process. However, healthy subjects showed stronger activation within the parietal lobe when processing information about probabilities and incentives. Parietal hypometabolism has frequently been described in PD patients with dementia [59,60] but also without cognitive impairments [61–63]. Some studies also report direct associations between hypometabolism within the parietal lobe and cognitive functions in PD patients [64, 65]. Additionally, less parietal activations in an fMRI Stroop task in PD patients on dopaminergic medication, compared to healthy subjects, were described by Fera et al. [34]. Whereas PD patients showed similar activation patterns within the PFC in the interference condition of the Stroop paradigm, healthy subjects showed an additional involvement of the superior parietal lobe. Even though in healthy subjects parietal and frontal brain regions strongly interact in the context of cognitive functions – specifically executive and attention functions (e.g. [66,67]) – PD patients of the current study are not impaired on the fMRI task despite reduced parietal activity. As discussed in our previous study on neural correlates of decision making in healthy subjects [25], we assume that the parietal lobe – beyond its relevance for attention and executive functions – might be associated with approximate arithmetic operations while comparing incentives and probabilities of the two concurring alternatives presented in each trial (see e.g. [68–70]). Potentially, deliberative strategies of PD patients differ from those of the normal subjects: less activation in this region might be due to neglecting information about incentives. Subjects knew that they would not receive the incentives presented. PD patients potentially pay less attention to the incentives to minimize interference susceptibility. The assumption that PD patients paid less attention to the incentives is also supported by the group comparison within the PD group. In contrast to the healthy subjects, PD patients did not show a significant BOLD increase when additional information about incentives was presented (compared to both control conditions). Thus, it might be that subjects based their decision on the same information in each condition (e.g. the number of dice in condition A and B that delivered information about probabilities) and ignored the incentive information provided in condition A.

We hypothesized that PD patients exhibit different activation patterns within the anterior cingulate gyrus. We suggest that cingulate activations within healthy subjects can be attributed to conflict detection mechanisms that are discussed in decision making [40,71, 72]. We did not find a significant activation difference between healthy subjects and patients in this region. However, the BOLD increase seen in healthy subjects when information about probabilities and incentives is given, compared to the control condition with probabilities but no incentives, was absent within the patient group. This again might be attributed to a difference in information processing. If PD patients ignored incentives provided in the activation condition, the conflict between options diminished. Ignoring incentives could be an efficient strategy in the decision process demanded in our fMRI task, as subjects could easily know that those alternatives with lower winning probabilities (alternatives including fewer dice) are the risky options. Further studies are needed to clarify whether PD patients are more willing to ignore information about incentives and, if so, why. One hypothesis to be tested is that PD patients pay less attention to incentives as a compensation strategy because, otherwise, their cognitive, deliberative decision process is prone to be affected by mere information about reward and punishment.

One limitation of our study is that we cannot determine the impact of dopaminergic treatment on the behavioural and imaging results because we did not compare patients 'on' and 'off' medication. Recent studies suggest a complex interaction of reward processing as well as cognitive functioning and dopaminergic treatment in PD patients, most likely following an inverted U-shaped function [73]. This relationship seems to be further influenced by disease severity [36]. Thus, PD patients are not necessarily reduced in reward processing and associated behaviour per se. Cools and co-workers reported that dopaminergic treatment can improve cognitive functions but concurrently boost impulsivity [74] (see also [75]). Thus, intact cognitive functions but risky decision making in terms of reward-seeking behaviour and neglecting feedback might be associated with the current dopaminergic state. The same seems to account for neuroimaging results in PD patients. The PET study by Cools and colleagues [32], in which patients with PD performed a spatial working memory task and a planning task, has shown that activation pattern in patients with normal dosage of dopamine treatment did not differ from healthy comparison subjects. However, when the same patients were off medication, they showed stronger PFC activation within the experimental condition and decreased activation within the control condition. Further studies are needed to clarify if the patterns of our fMRI results in PD patients are specific for PD patients on medication and might be different in PD patients without dopaminergic treatment. As some finding suggest that impulsive or risk seeking behaviour may be stronger related to dopamine agonist treatment compared to levodopa therapy (see Introduction), it would also be of interest to investigate the specific impact of the type of medication on decision making processes in PD patients.

Another limitation is the fact that the subjects performed the GDT outside the scanner whereas the non-feedback task is performed within the scanner. Although the patients' performance in the non-feedback task is not impaired and not different form the healthy subjects' performance, we cannot exclude that environmental factors such as the scanner's noise somehow affect task performance. In future studies, performing both tasks in the same setting (e.g. within the scanner) would be a better methodology.

## Acknowledgement

This work was supported by the German Research Foundation (BR 2894/1-1 and BR 2894/4-1). We thank Dr. Renate Husmann for her support in recruiting the patients for this study. We also thank all patients and healthy subjects for participating.
